# The Willingness to Donate Organs in Medical Students From an International Perspective: A Meta-Analysis

**DOI:** 10.3389/ti.2022.10446

**Published:** 2022-06-28

**Authors:** Marina Iniesta-Sepúlveda, Ana I. López-Navas, Pedro R. Gutiérrez, Pablo Ramírez, Antonio Ríos

**Affiliations:** ^1^ Department of Psychology, Catholic University of Murcia, UCAM, Murcia, Spain; ^2^ Department of Surgery, University of La Laguna (ULL), San Cristóbal de La Laguna, Spain; ^3^ Department of Surgery, Pediatrics, Obstetrics y Gynecology, University of Murcia, Murcia, Spain; ^4^ Transplant Unit, Surgery Service, IMIB – University Clinical Hospital Virgen de la Arrixaca, Murcia, Spain

**Keywords:** meta-analysis, willingness, medical students, organ donation, cultural

## Abstract

Attitude toward organ donation mobilizes donation behavior and makes transplant surgery possible. As future health professionals, medical students will be a relevant generating opinion group and will have an important role in the organ requesting process. The goals of this meta-analysis were to obtain polled rates of medical students who are in favor, against, or indecisive toward cadaveric organ donation in the studies conducted around the world, and to explore sociocultural variables influencing the willingness to donate. Electronic search and revision of references from previous literature allowed us to locate 57 studies fulfilling the inclusion criteria. Data extraction and risk of bias assessment were performed by two independent investigators. Pooled estimations were computed assuming a random-effects model. Despite the fact that willingness to donate was elevated in medical students, estimated rates in studies from different geographical areas and sociocultural backgrounds exhibited significant differences. The age and the grade of the students also influenced the rate of students in favor. Donation campaigns should take into account cultural factors, especially in countries where certain beliefs and values could hamper organ donation. Also, knowledge and skills related to organ donation and transplant should be acquired early in the medical curriculum when a negative attitude is less resistant to change.

## Introduction

Despite the advances in the field of organ donation and transplant, current rates of donation are still insufficient to cover minimum needs. The organ deficit is the main cause of death in waitlisted patients (1). There are several factors involved in the process of requesting and donating organs for transplants. Sociocultural factors are one of the main sources of variability among studies on the attitudes toward donation. First, geographical area influences the willingness to donate. Differences in organ donation systems and organ requesting protocols in each country mean that even people from similar cultural backgrounds (e.g., Latin) and living in different geographic areas could exhibit different levels of disposition to donate (2). Second, attitudes to donation are dependent on the local cultural and socioeconomic background. Death conceptions, religion, and values must be considered by the organ donation system in each country for transplantation programs to be successful (3, 4). Finally, sociodemographic factors such as age, gender, and educative level have also been shown to have influence on attitudes toward donation and transplant.

Health professionals have an important role in the successful development of the organ donation process (5). In the community context, they are one of the most relevant opinion-generating groups. Moreover, negative attitudes based on information provided by professionals are more resistant to change since they are supported by experts (6). Medical students are the new generation of clinicians, and therefore, the future link between donors and recipients.

Obtaining knowledge about attitudes toward cadaveric organ donation in medical students has been considered of particular importance and exists in a wide range of scientific literature. Research has been conducted in different countries and cultural backgrounds, has examined different dimensions of organ donation attitudes (awareness, willingness, registration, etc.), and has used a variety of methodological procedures. As a consequence, the results reported a high heterogeneity across studies. Despite its extension, the literature has not been systematically integrated and factors behind the heterogeneity of findings have not been explored yet. Meta-analytical procedures could contribute to reaching well-established conclusions about the intention of medical students to donate their organs after death.

Following the PICOS strategy to formulate questions in meta-analyses, the current study intended to answer the following question: what is the rate of medical students (participants) who are in favor (outcome) of donating their organs after death (intervention) in observational studies (study design)? From this question, two goals were considered: 1) to obtain the polled estimated rate of medical students who were in favor, against, or indecisive toward cadaveric organ donation; and 2) to explore sociocultural variables influencing the willingness to donate. We expected that the elevated pooled rate of medical students in favor of cadaveric donation would be superior to rates of students against and indecisive. It is likely that rates of students willing to donate were influenced by potential moderators, such as geographical area, grade of students, and gender.

## Materials and Methods

This meta-analysis was performed following the PRISMA 2020 Guideline for Reporting Meta-analyses (7) and the MOOSE Checklist for Meta-analyses of Observational Studies (8). See Supplementary Data Sheet S1.

### Inclusion and Exclusion Criteria

To be included in the meta-analysis studies had to fulfill the following eligibility criteria: 1) assess willingness to donate organs after death; 2) report necessary statistics to compute the proportion of participants who are willing to donate (events and sample size); 3) participants were medical students; 4) observational designs without experimental manipulations; and 5) published in English, Spanish, or Portuguese. Studies examining attitudes toward living donation, donation of specific organs, studies that did not report results for medical students separately from samples of other populations (e.g., non-medical students, general public, etc.), and studies sharing samples (totally or partially) with other included studies were excluded. Studies in languages other than English, Spanish, or Portuguese could not be included due to the language limitations of researchers.

### Search Strategy

**FIGURE 1:**
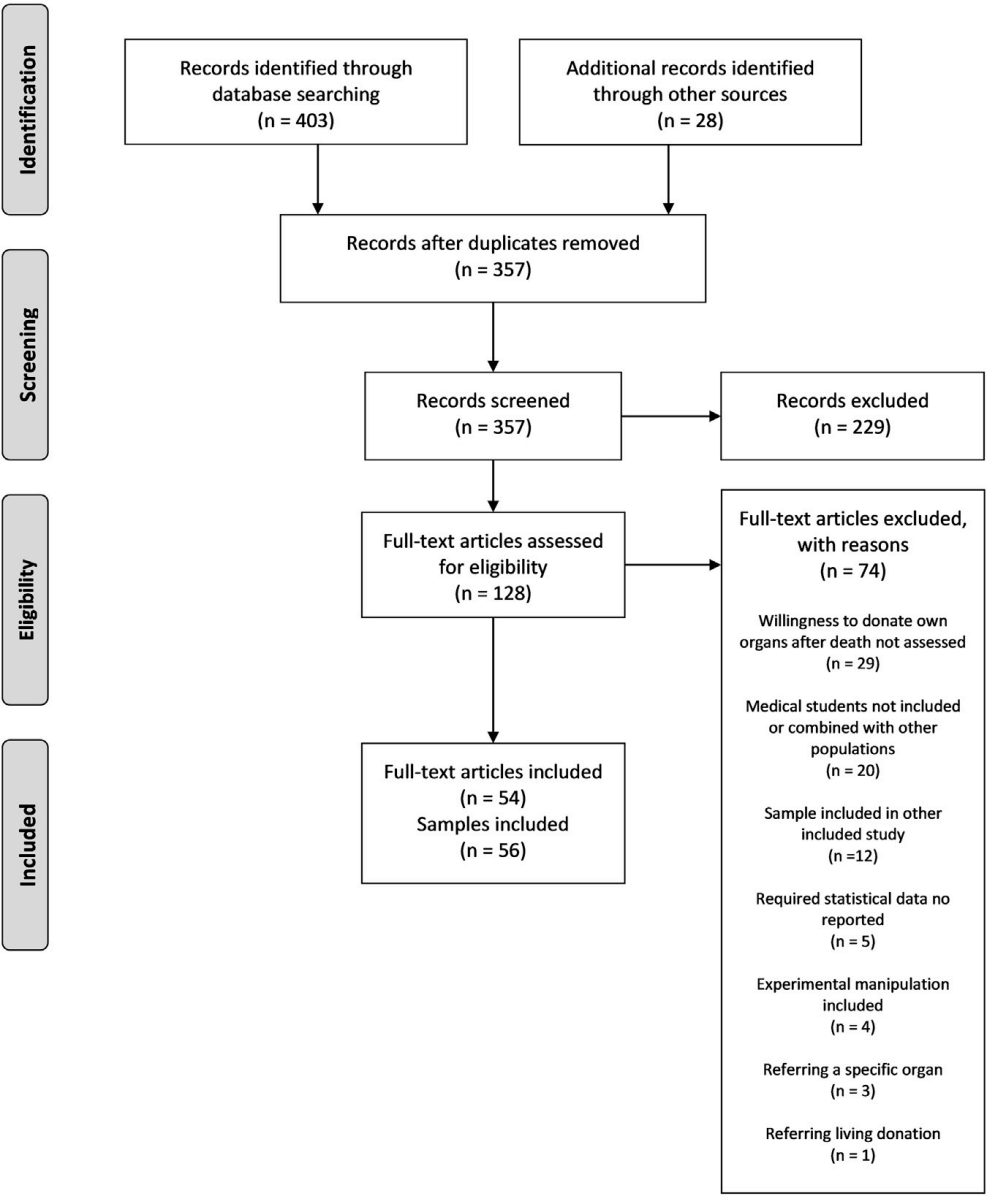
PRISMA flow diagram.

An electronic search was conducted in PubMed, CINALH Complete, PsycInfo, and Psychology and Behavioral Sciences Collection until February 2021. English and Spanish keywords were organ donation AND (attitude OR willingness OR perceptions OR beliefs OR opinions) AND medical students. References of previous meta-analyses (9–11) and studies collected were also screened. Finally, the most prolific authors in the field were contacted to request potential unpublished data. Figure 1 shows the search and eligibility processes in the PRISMA flow diagram.

The electronic search yielded 403 outputs, and 28 references were located from previous publications. After deleting duplicates, the title and abstract of 357 papers were reviewed. After excluding a further 229, the full text of 128 articles was reviewed to assess their potential inclusion; 73 articles were rejected due to reasons shown in Figure 1. Finally, 54 papers (12–65) including 56 separate samples fulfilled the inclusion criteria.

### Data Extraction

A data extraction protocol including statistics and potential moderator variables was elaborated and applied by two independent investigators to each selected study. Variables concerning participants were: 1) gender (percentage of men); 2) rate of men in favor; 3) rate of women in favor; 4) mean age; 5) the percentage of students in each grade; 6) proportion of first-grade students in favor; 7) proportion of students in the last grade in favor; 8) country of participants; 9) continent; 10) cultural background in the country of participants; and 11) the percentage of participants of each religion. Variables related to the methodology of studies were: 1) year of survey; 2) completion rate; 3) type of measure (interview or self-report); 4) administration modality (face-to-face, online, or both); and 5) methodological quality of the study (rated from 0 to 5, see Quality Assessment section).

### Risk of Bias Assessment

To assess the risk of bias in individual studies, a five-item checklist was elaborated based on the STROBE Checklist for cross-sectional studies (66). Items were rated as follows: 1) setting: whether the study provided information about locations, setting, and dates of data collection (1 yes, 0 no); 2) sample size: whether the study explained how the sample size was arrived at (1 yes, 0 no); 3) participants: whether the study reported eligibility criteria and methods of selection of participants (1 yes, 0 no); 4) completion rate: whether the study reported the percentage of distributed surveys that were retrieved (1 yes, 0 no); and 5) outcome: whether the study employed a validated outcome measure or conducted a pilot study prior to its administration (1 yes, 0 no). A methodological quality score was computed as the sum of the five items.

### Statistical Analysis

The primary outcome was the pooled estimate rate (proportion) of medical students who were willing to donate organs after death. Rates of students against and indecisive were also extracted as secondary outcomes. Under the assumption that samples of selected studies could be representative of different populations, pooled rates were computed assuming a random-effects model, where each individual proportion was pondered by its precision. Heterogeneity was examined by computing *Q* statistics and the percentage of the observed variance between studies’ *I* ^*2*^. To analyze the effect of potential moderator variables on the primary outcome (rate of students in favor), ANOVAs with *Q*_*B*_ statistics and meta-regression models with *Q*_*R*_ statistics were computed for categorical and continuous variables, respectively. The percentage of explained variance was assessed by *R* ^*2*^ index (67). Publication bias analysis included the Egger test and the construction of a funnel plot implementing the trim-and-fill method (68). All data analyses were conducted in Comprehensive Meta-Analysis (CMA) 3.0 (69).

## Results

### Study Characteristics and Risk of Bias

Table 1 shows the main characteristics of the 56 independent studies included in the meta-analysis. Studies were conducted in 25 different countries between 1999 and 2020. The total sample included 33,536 medical students with mean ages between 17.60 and 26.35 years. The percentage of men ranged from 16.6% to 93.8%. The completion rate reported by the studies ranged from 32% to 100%. Concerning the risk of bias, the mean methodological quality was 2.18, with 35.1% of studies having scores ≥3 See Supplementary Table S1.

**TABLE 1:** Summary of the included studies.

Study	Year of survey	Country	No. of participants	Completion rate, %	Quality, range 1–5	Age, mean	Men, %	In favor, %	Against, %	Indecisive, %
Akkas et al. (12)	2013	Turkey	100	66.80	3	17.60	43.0	54.00	16.00	30.00
Akkas et al. (12)	2013	Turkey	100	66.80	3	24.20	56.0	70.00	14.00	16.00
Ali et al. (13)	2011	Pakistan	158	81.02	3	20.00	36.7	44.94	—	—
Alnajjar et al. (14)	2019	Saudi Arabia	113	74.83	5	20.04	93.8	55.75	8.85	35.40
AlShareef et al. (15)	2016	Saudi Arabia	225	36.12	2	22.77	68.0	38.22	19.11	42.67
Anwar et al. (16)	2019	Bangladesh	100	—	1	—	—	28.00	16.00	48.00
Ashfaq et al. (17)	2017	Pakistan	400	—	3	20.98	50	61.25	—	—
Atamañuk et al. (18)	2016	Argentina	1012	96.80	3	21.40	35.5	81.92	—	—
Bilgel et al. (19)	—	Turkey	409	80.50	2	20.30	49.9	58.44	22.74	18.83
Burra et al. (20)	—	Italy	100	51.30	1	23.70	29.0	88.00	—	—
Cahill & Ettarh (21)	2007	Ireland	187	87.00	2	—	—	63.64	7.49	28.88
Chung et al. (22)	2006	China	655	94.00	2	21.00	58.0	85.04	—	—
Dahlke et al. (23)	—	Germany	165	—	1	21.50	35.2	56.36	—	—
Dahlke et al. (23)	—	Japan	99	—	1	22.40	72.7	52.53	—	—
Dahlke et al. (23)	—	United States	66	—	1	23.90	48.5	65.15	—	—
Dibaba et al. (24)	2019	Ethiopia	320	—	2	23.48	57.8	58.12	—	—
Dutra et al. (25)	2002	Brazil	779	77.82	2	21.90	59.5	69.06	30.68	—
Edwards et al. (26), Essman (29)	2005	United States	500	93.00	3	24.00	50.0	82.40	5.00	9.00
El-Agroudy et al. (27)	2017	Bahrein	376	75.20	2	22.10	39.1	71.81	18.88	11.97
Englschalk et al. (28)	2015	Germany	181		2	23.10	37.6	82.32	7.18	9.94
Figueroa et al. (30)	2011	Holland	506	84.00	3	20.76	26.6	79.84	5.73	14.03
Galvao et al. (31)	—	Brazil	347	32.00	3	—	—	89.91	10.09	—
Goz et al. (32)	—	Turkey	213	36.91	2	—	—	56.81	—	—
Hamano et al. (33)	2018	Japan	702	100.00	2	25.00	—	54.70	13.96	31.05
Hasan et al. (34)	2019	Pakistan	157	82.00	2	20.60	16.6	41.40	—	—
Inthorn et al. (35)	2009	Germany	466	95.10	2	—	—	63.52	—	—
Jamal et al. (36)	2017	Pakistan	150	88.50	4	—		61.33	—	—
Jung et al. (37)	—	Romania	140	—	0	20.50	30.0	81.43	3.57	15.00
Kirimlioglu et al. (38)	—	Turkey	214	71.30	2	20.00	45.8	22.43	27.10	—
Kobus et al. (39)	—	Poland	203	—	0	21.80	-	94.58	—	—
Kocaay et al. (40)	2013	Turkey	88	—	1	—	—	60.23	—	—
Kozlik et al. (41)	2012	Poland	400	—	2	21.80	37.3	90.50	3.00	6.50
Lei et al. (42)	2016	China	284	—	2	—		15.14	—	—
Lima et al. (43)	2007	Brazil	300	85.70	3	—	51.0	62.00	—	—
Liu et al. (44)	2019	China	1363	90.90	2	21.5	39.5	62.73	37.27	
Marques et al. (45)	2008	Puerto Rico	227	76.70	3	—	49.1	88.55	11.01	—
Marván et al. (46)	2018	Mexico	205	—	3	—	48.3	91.71	—	—
Mekahli et al. (47)	2006	France	571	—	1	18.50	34.5	81.09	13.49	5.43
Naçar et al. (48)	2014	Turkey	464	94.70	1	20.90	48.9	50.00	5.82	44.18
Najafizadeh et al. (49)	2006	Iran	41	—	1	22.80	44.0	87.80	4.88	—
Ohwaki et al. (50)	2004	Japan	388	100.00	2	—	74.0	59.02	15.98	21.91
Ríos et al. (51)	2011	Spain	9275	95.70	5	21.00	28.2	79.53	1.66	18.91
Rydzewska et al. (52)	—	Poland	569	—	0	21.77	25.8	92.97	2.46	4.57
Sağiroğlu et al. (53)	2012	Turkey	356	71.80	2	20.40		49.44	16.85	33.71
Sahin and Abbasoglu (54)	2013	Several countries	1541	—	2	21.80	41.0	94.35	1.36	4.28
Sampaio et al. (55)	—	Brazil	518	49.01	1	—	25.9	84.94	1.35	13.71
Sanavi et al. (56)	2008	Iran	262	97.00	1	22.10	32.0	85.11	—	—
Sayedalamin et al. (57)	2014	Saudi Arabia	481	—	2	21.39	48.0	31.81	68.19	—
Sebastián-Ruiz et al. (58)	2015	Mexico	3056	—	2	20.30	53.3	73.99	26.01	—
Tagizadieh et al. (59)	2016	Iran	400	—	2	26.35	59.0	85.00	15.00	—
Tuesca et al. (60)	1999	Colombia	993	84.27	5	25.00	52.6	84.79	6.65	8.56
Tumin et al. (61)	2014	Malaysia	264	88.00	4	—	—	72.73	—	—
Verma et al. (62)	—	India	1463	73.00	3	-	44.9	65.62	34.38	—
Wu et al. (63)	—	China	264	88.00	3	20.25	29.5	39.77	42.05	18.18
Zahmatkeshan et al. (64)	2012	Iran	340	—	3	—	—	79.12	9.41	11.47
Zhang et al. (65)	—	China	199	—	1	—	43.2	32.16	27.14	40.70

### Pooled Rates of Medical Students in Favor, Against, and Indecisive

Table 2 shows combined estimated proportions and confidence intervals for each outcome in the meta-analysis. In the primary outcome, a combined percentage of 69.2% (95% CI: 64.7%–73.4%) of medical students was willing to donate their organs after death. Significant and high heterogeneity was observed (*I* ^*2*^ = 98.25%). Regarding secondary outcomes, the pooled estimation of students against donating, including 36 studies, was 11.7% (95% CI: 8.4%–16.1%) and the pooled estimation for indecisive students, including 27 studies, was 17.7% (95% CI: 14%–22%). Heterogeneity tests showed significant and high variability among studies in both against (*I* ^*2*^ = 98.82%) and indecisive (*I* ^*2*^ = 97.33%) participants.

**TABLE 2:** Pooled estimated rates, confidence intervals, and heterogeneity indexes for study outcomes.

Outcome	*K*	*Q*	*I* ^*2*^	*p*_+_	95% C.I.	
					*l*_l_	*l*_u_
Students in favor	56	3144.31***	98.25	0.692	0.647	0.734
Students against	36	2978.40***	98.82	0.117	0.084	0.161
Indecisive students	27	973.39***	97.33	0.177	0.140	0.220

C.I., confidence interval; k, number of studies; Q, heterogeneity statistic; I^2^, heterogeneity index; *p*_+_, pooled estimated rate, l_I_ and l_u_, lower and upper confidence limits.

***p < 0.001.

### Factors Influencing the Willingness to Donate

#### Participant-Related Variables

##### Continent, Culture, and Religion

**FIGURE 2:**
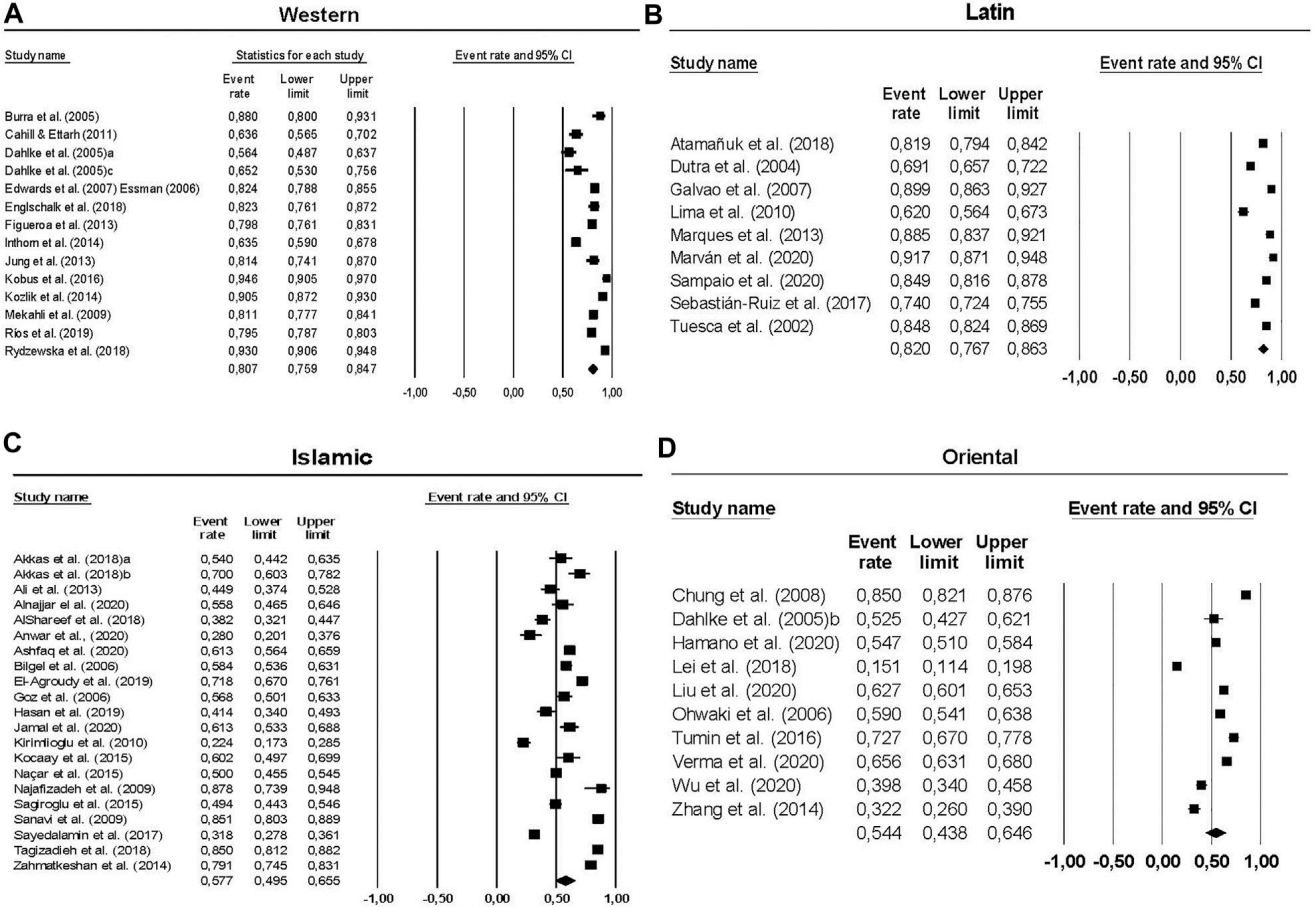
Forest plots of individual rates and confidence intervals for each study (squares) and pooled estimations and confidence intervals for each cultural background (diamonds). **(A)** Forest plot of individual and pooled rates of students willing to donate in Western countries. Individual rates vary from 0.564 to 0.940. The pooled estimated rate by the random-effects model was 0.807. **(B)** Forest plot of individual and pooled rates of students willing to donate in Latin countries. Individual rates vary from 0.620 to 0.917. The pooled estimated rate by the random-effects model was 0.820. **(C)** Forest plot of individual and pooled rates of students willing to donate in Islamic countries. Individual rates vary from 0.224 to 0.878. The pooled estimated rate by the random-effects model was 0.577. **(D)** Forest plot of individual and pooled rates of students willing to donate in Oriental countries. Individual rates vary from 0.151 to 0.850. The pooled estimated rate by the random-effects model was 0.544.

Significant differences were observed depending on the continent where the study was conducted (*Q*_3_ = 27.13, *p* <0.000). The highest pooled rates of students in favor were obtained by the studies conducted in North America (*k* = 2, *p*_*+*_ = 0.753, 95% CI [0.554, 0.882]), Latin America (*k* = 9, *p*_*+*_ = 0.820, 95% CI [0.767, 0.863]), and Europe (*k* = 20, *p* = 0.718, 95% CI [0.642, 0.784]) which were significantly superior to the pooled rate for studies in Asia (*k* = 23, *p*_*+*_ = 0.580, 95% CI [0.503, 0.654]). Given these results, and to obtain a more accurate view of differences, we considered grouping studies by predominant culture in the country of participants. Figure 2 shows forest plots of pooled estimations for each cultural background and individual rates for each study. Cultural background significantly influenced the willingness to donate (*Q*_3_ = 49.850, *p* < 0.000). Higher rates were observed for studies in countries with Latin (*k* = 9, *p*_*+*_ = 0.820, 95% CI [0.767, 0.863]) and Western (*k* = 14, *p*_*+*_ = 0.807, 95% CI [0.760, 0.850]) cultural backgrounds, finding significant differences with Islamic (*k* = 21, *p*_*+*_ = 0.577, 95% CI [0.495, 0.655]) and Oriental (*k* = 10, *p*_*+*_ = 0.544, 95% CI [0.438, 0.646]) countries. Regarding religion, the percentage of Catholic students showed a positive and significant relationship with the proportion of students in favor (*k* = 15, *b*_*j*_ = 0.02, *Q*_1_ = 28.09, *p* <0 .000, *R* ^2^ = 0.44) whereas the percentage of Muslim students was not related to the rate of students in favor (*k* = 10, *b*_*j*_ = −0.01, *Q*_1_ = 2.13, *p* = 0.144, *R* ^2^ = 0.00). The influence of the percentage of students affiliated with other religions could not be analyzed due to the reduced number of studies that reported these data.

##### Age and Grade of Participants

The mean age of participants showed a significant and positive relationship with the proportion of students in favor of donating (*k* = 39, *b*_*j*_ = 0.16, *Q*_1_ = 4.85, *p* = 0.024, *R* ^2^ = 0.10) explaining 10% of the variance. Results of meta-regression analyses showed that percentages of students in 2nd, 3rd, 4th, 5th, and 6th grade included in the studies, were not significant predictors of the willingness to donate (*p* >0 .05). Only the percentage of first-grade students showed a significant and negative relationship with the proportion of students in favor of donation (*k* = 25, *b*_*j*_ = -0.01, *Q*_1_ = 4.75, *p* = 0.029, *R* ^2^ = 0.06) with 6% of the accounted variance. There were marginally significant differences between first-grade (*k* = 13, *p*_*+*_ = 0.65, 95% CI [0.55, 0.73]) and sixth-grade students (*k* = 10, *p*_*+*_ = 0.79, 95% CI [0.67, 0.87]) according to the subgroup analysis (*Q*_1_ = 3.79, *p* = 0.052).

##### Gender

The percentage of men was not a significant predictor of the willingness to donate (*k* = 43, *b*_*j*_ = −0.02, *Q*_1_ = 2.56, *p* = 0.11, *R* ^2^ = 0.00). Similarly, subgroup analysis did not yield significant differences (*Q*_1_ = 1.487, *p* = 0.223) in the proportion of men (*k* = 9, *p*_*+*_ = 0.61, 95% CI [0.52, 0.69]) and women (*k* = 9, *p*_*+*_ = 0.68, 95% CI [0.59, 0.77]) in favor.

#### Methodological Variables

Meta-regression analysis revealed that the completion rate (*k* = 34, *b*_*j*_ = 0.00, *Q*_1_ = 0.02, *p* = 0.900, *R* ^2^ = 0.00) and the methodological quality score (*k* = 56, *b*_*j*_ = −0.03, *Q*_1_ = 0.06, *p* = 0.810, *R* ^2^ = 0.00) were not significantly associated with the proportion of students willing to donate. Only the year of survey (*k* = 41, *b*_*j*_ = −0.07, *Q*_1_ = 8.79, *p* = 0.003, *R* ^2^ = 0.08) was negatively associated with the rate of students in favor. There were not significant differences between face-to-face (*k* = 48, *p*_*+*_ = 0.68, 95% CI [0.64, 0.73]) and online (*k* = 6, *p*_*+*_ = 0.68, 95% CI [0.46, 0.85]) administration (*Q*_1_ = 0.000, *p* = 0.997).

### Publication Bias Analysis

**FIGURE 3:**
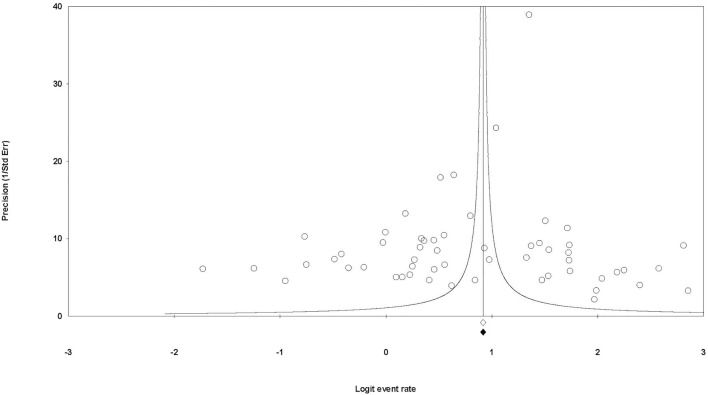
Funnel plot of the individual observed rates for each study (circles) and observed (white diamond) and adjusted (black diamond) pooled rates of students willing to donate. The absence of imputed values to achieve symmetry in the dots’ distribution and the equivalence between observed and adjusted pooled rates allow for us to discard publication bias.

First, results from Egger’s test were not significant (*b*_*0*_ = −2.89; *t*[54] = 1.60, *p* = 0.115), supporting the absence of publication bias. Second, after the implementation of the trim-and-fill method, it was not necessary to introduce imputed values into the funnel plot to reach symmetry (Figure 3), with the pooled proportion of adjusted values equal to the pooled proportion of observed values.

## Discussion

This is the first meta-analysis on the willingness to donate in medical students. Similarly, this is the first work analyzing cultural and individual variables as potential explaining factors of the variability of results reported by studies around the world. Results have revealed a pooled rate of close to 70% of students willing to donate their organs after death. This is higher than the observation in studies conducted with the general public in different countries (10, 70–72) supporting that medical students have a heightened awareness of organ donation, similar to students from other health disciplines (32, 73, 74).

However, results in primary studies exhibited high heterogeneity, pointing to the presence of factors influencing willingness to donate. Both geographical area (continent) and cultural background had significant effects on the rate of students in favor. Studies conducted in countries with Latin (82%) and Western (70.6%) cultures obtained the greatest percentages, followed by Islamic countries (57.7%) and studies in countries with an Oriental culture (54.4%) which obtained the lowest percentage. These results are in line with previous literature. The meta-analysis by Mekkodathil et al. (10), including studies with the general public from Islamic countries, reported a pooled percentage of favorable attitude toward donation of less than 50%. Also, studies conducted with Asian populations have reported reduced rates of donation intention and registration among students, health workers, and the general public (75).

Sociocultural background includes social, spiritual, religious, and family beliefs and values that affect the decision-making process about donation. Regarding medical students in Islamic countries, motives related to body preservation after death were reported by students against donating their organs in some included studies (15, 19, 49, 54, 65). Conversely, the percentage of students worried about the mutilation of the body after death was considerably low in studies conducted in Western (30, 52) and Latin (59) cultural backgrounds. As in Western (26, 30, 40, 52, 76) or Latin countries (31, 59, 61) religious motives against donation were reported by reduced percentages of medical students in studies conducted in Turkey (32, 39, 49, 54). However, knowing the attitude toward donation and transplant promoted by participants’ own religion can influence individual attitudes. In some included studies conducted in Saudi Arabia, Turkey, and Iran, about 30% of medical students ignored whether religion was in favor of donation and transplant (15, 41, 60). By contrast, in countries with high predisposition rates such as Spain, only 12% of medical students did not know their religion’s posture on donation and transplant (52).

In countries with a predominant Oriental culture, family opinion about donation seemed to be of particular importance. In the study by de Ohwaki et al. (51), more than 65% of medical students stated that their families would disagree with organ donation. Similarly, Lei et al. (43) observed that 95.5% of the students with no favorable attitude believed that their family was against donation. Oriental culture confers to family a relevant role in the life of individuals. Traditional values emphasized family interests over the individual’s ones (43). Although in a Western or Latin cultural context, family’s opinion influences the willingness to donate (52), the percentages of students who had discussed donation with their family (60%–70%) were considerably elevated (18, 26, 52, 59). Also in these countries, it has been reported that elevated proportions of medical students think that their parents’ opinion is favorable (52, 59). Therefore, the family would play a beneficial role to promote favorable attitudes in Western and Latin cultural contexts. The importance of body preservation is another factor that affects the intention to donate after death in Asian medical students. A high percentage of students recognized concerns about body mutilation in the organ extraction process in some studies (22, 43). The Confucian heritage that promotes the idea of body care as a way of respect to parents, together with beliefs related to life after death, contributes to the importance of body preservation after death in Oriental cultures (75). As commented, the importance of body preservation was not a relevant reason against donation in cultural contexts with high rates of willingness to donate, being more rated than other motives such as the lack of information (26, 52, 59) and fear of trafficking or fair organ allocation (26, 52, 59).

According to the reports from the Global Observatory on Donation and Transplantation (77) in 2020, cultural differences observed in willingness to donate could be reflected by the rates of deceased donors in the countries of studies included in this meta-analysis. Using the same classification by cultural background, the highest mean of deceased donors per million population was observed in Western countries (16.38), followed by the mean in Latin (7.40), Islamic (3.86), and Oriental (1.69) countries. As it can be seen, the trend was similar to the observed willingness to donate, except for Latin countries, in which despite having an elevated rate of students in favor in this meta-analysis, the rates of deceased donors were discrete and lower than in Western countries. Possible explanations for this difference are that medical students were not representative of the general population in Latin America and that in addition to the attitudes, there were other variables (economic, related to donation system, etc.) influencing the factual deceased donor rates.

Age was positively related to the rate of students in favor. Given that the population studied in this meta-analysis was medical students, whose level of knowledge rises yearly, it is highly probable that the change in their perspective would be due to the educational level more than to the age effect itself. In fact, the percentage of first-grade students included had a negative impact on the proportion of students in favor. Moreover, the subgroup analysis revealed differences between first- (65%) and sixth-grade students (79%). Taken together, these results may support the positive influence of years of training received by the students on their willingness to donate. It has been demonstrated that knowledge about aspects related to donation and transplant has a positive impact on attitudes toward donation (30, 52, 78). In addition, students in more advanced grades could have more opportunities for contact with transplant patients and donors or have attended campaigns or workshops to promote awareness toward donation. These experiences have also shown beneficial effects on the attitude to donation (18, 52).

In this meta-analysis, gender was not significantly related to the rate of students in favor, whereas individual studies have shown contradictory findings: existing studies where women exhibited a more favorable attitude (19, 32, 52) and studies where significant differences were not observed (27). Despite the fact that our findings revealed a higher rate for women (68%) than for men (61%), the reduced number of subgroups included in the analysis could explain the absence of significant differences.

Regarding methodological variables, the completion rate did not affect the rate of students willing to donate. Percentage of response could be a risk of bias indicator in attitudinal studies since higher participation could be associated with greater interest in the topic, or even with a more favorable attitude. As a consequence, it would be desirable that at least 75% of spread surveys could be included in the analysis (78). In this meta-analysis, 80% of studies that reported the completion rate showed percentages over 70%. This fact could explain the absence of significant effects on the willingness to donate. Remarkably, 39% of the included studies did not report the completion rate. The modality of administration of surveys (face-to-face vs. online) also affected the rate of students in favor, when taking into account that only six studies used online surveys. Finally, the year in which the survey was conducted showed an inverse association with the rate in favor, pointing to the absence of an increasing trend in the willingness to donate through the years.

The findings of this meta-analysis must be interpreted attending to some limitations. First, some of the studies included presented low scores in methodological quality assessment. The absence of sample size estimation procedures, the absence of random sampling, and the use of non-validated measures were the main weaknesses in the included studies. This could lead to bias in sample representativeness, and variability in the measurement of the willingness to donate. Despite this, it is remarkable that neither the risk of bias nor other methodological variables had a significant impact on the rate of students in favor. Second, all studies used self-report measures. Therefore, inherent disadvantages to self-reports in attitudinal studies (e.g., the trend to answer in a socially desirable way) could affect our results. Third, relevant variables such as discussing organ donation with family, contact with patients and donors, and frequency of other altruistic activities could not be analyzed as influencing factors because they were not reported by enough studies.

Despite these limitations, these results suggest practical implications for medical curriculum design. According to our findings, medical students present a high willingness to donate their organs, improving their attitudes as they progress in their medical careers. However, the percentage of students against and indecisive is still considerable. This picture is heterogeneous around the world, in which there are remarkable differences depending on the sociocultural background which students are immersed. This meta-analysis has evidenced that countries with Oriental and Islamic cultures showed the lowest rates of medical students willing to donate their organs after death. As commented, these studies have shown that the major reasons behind poor donation rates are cultural-related myths, lack of information, and religious misconceptions. In recent years, some countries in these cultural backgrounds have made efforts to include organ donation and transplantation contents in the medical curriculum. However, these modifications have been mainly focused on the acquisition of knowledge (brain death concept, organ donation system functioning, waitlists, etc.) ignoring the approach to sociocultural and religious issues (79). In order to address cultural issues in the medical curriculum, the following aspects are considered of particular importance: 1) promoting the discussion of the topic with family, 2) providing information about the local religion’s attitude to donation, 3) discussing cultural-related death conceptions, and 4) providing reliable information about body manipulations in the donation process. Besides addressing cultural barriers, the possibility of taking advantage of certain cultural values to promote organ donation has been highlighted, for example, the Confucian values of helping others and positive life attitude in Chinese society (80). Knowledge and skills related to organ donation and transplant should be addressed early (first years) in the medical curriculum. This allows for saving resources from campaigns in medical professionals whose negative attitude is more resistant to change (6).

Given that the development of culture-specific campaigns and study plans implies being aware of beliefs, values, and practices of different population groups, future research should examine more deeply culture‐bound conceptualizations of death, organ donation, and other related aspects. Moreover, recommendations for the medical curriculum could be extrapolated to other relevant population targets, especially in educative contexts. This would be the case for adolescents, who are immersed in the development of their own system of values and attitudes.
