# Erratum: The Willingness to Donate Organs in Medical Students From an International Perspective: A Meta-Analysis

**DOI:** 10.3389/ti.2022.10820

**Published:** 2022-08-25

**Authors:** Transplant International Production Office

**Affiliations:** Frontiers Media SA, Lausanne, Switzerland

**Keywords:** meta-analysis, willingness, medical students, organ donation, cultural

The original article was incorrectly published as a “Review” article. The correct article type is “Meta-Analyses and Systematic Reviews.”

The publisher apologizes for this mistake.

