# Supplementary material for: The Willingness to Donate Organs in Medical Students From an International Perspective: A Meta-Analysis
Source: Transpl Int. 2022 Jun 28;35:10446. doi: 10.3389/ti.2022.10446 (PMC9273723; doi:10.3389/ti.2022.10446)
Supplement: Supplementary file 1 [file Table1.docx]

**Supplementary Material**

**Table. Quality Assessment of Studies**

| **Study** | **Setting** | **Sample size** | **Participants** | **Completion Rate** | **Outcome** | **Quality Range 1-5** |
| --- | --- | --- | --- | --- | --- | --- |
| Akkas et al. (2018) | 0 | 1 | 0 | 1 | 1 | 3 |
| Ali et al. (2013) | 0 | 1 | 1 | 1 | 0 | 3 |
| Alnajjar et al. (2020) | 1 | 1 | 1 | 1 | 1 | 5 |
| AlShareef et al. (2018) | 0 | 1 | 0 | 1 | 0 | 2 |
| Anwar et al. (2020) | 0 | 0 | 1 | 0 | 0 | 1 |
| Ashfaq et al. (2020) | 1 | 1 | 1 | 0 | 0 | 3 |
| Atamañuk et al. (2018) | 0 | 1 | 0 | 1 | 1 | 3 |
| Bilgel et al. (2006) | 0 | 0 | 0 | 1 | 1 | 2 |
| Burra et al. (2005) | 0 | 0 | 0 | 1 | 0 | 1 |
| Cahill & Ettarh (2011) | 0 | 1 | 0 | 1 | 0 | 2 |
| Chung et al. (2008) | 0 | 1 | 0 | 1 | 0 | 2 |
| Dahlke et al. (2005)a | 0 | 1 | 0 | 0 | 0 | 1 |
| Dahlke et al. (2005)b | 0 | 1 | 0 | 0 | 0 | 1 |
| Dahlke et al. (2005)c | 0 | 1 | 0 | 0 | 0 | 1 |
| Dibaba et al. (2020) | 1 | 1 | 0 | 0 | 0 | 2 |
| Dutra et al. (2004) | 0 | 1 | 0 | 1 | 0 | 2 |
| Edwards et al. (2007) Essman (2006) | 0 | 1 | 0 | 1 | 1 | 3 |
| El-Agroudy et al. (2019) | 0 | 1 | 0 | 1 | 0 | 2 |
| Englschalk et al. (2018) | 0 | 1 | 0 | 0 | 1 | 2 |
| Figueroa et al. (2013) | 0 | 1 | 0 | 1 | 1 | 3 |
| Galvao et al. (2007) | 0 | 1 | 1 | 1 | 0 | 3 |
| Goz et al. (2006) | 0 | 0 | 0 | 1 | 1 | 2 |
| Hamano et al. (2020) | 0 | 1 | 0 | 1 | 0 | 2 |
| Hasan et al. (2019) | 1 | 0 | 0 | 1 | 0 | 2 |
| Inthorn et al. (2014) | 0 | 0 | 0 | 1 | 1 | 2 |
| Jamal et al. (2020) | 0 | 1 | 1 | 1 | 1 | 4 |
| Jung et al. (2013) | 0 | 0 | 0 | 0 | 0 | 0 |
| Kirimlioglu et al. (2010) | 0 | 1 | 0 | 1 | 0 | 2 |
| Kobus et al. (2016) | 0 | 0 | 0 | 0 | 0 | 0 |
| Kocaay et al. (2015) | 0 | 1 | 0 | 0 | 0 | 1 |
| Kozlik et al. (2014) | 1 | 1 | 0 | 0 | 0 | 2 |
| Lei et al. (2018) | 0 | 1 | 0 | 0 | 1 | 2 |
| Lima et al. (2010) | 0 | 1 | 0 | 1 | 1 | 3 |
| Liu et al. (2020) | 0 | 1 | 0 | 1 | 0 | 2 |
| Marques et al. (2013) | 0 | 1 | 0 | 1 | 1 | 3 |
| Marván et al. (2020) | 1 | 1 | 0 | 0 | 1 | 3 |
| Mekahli et al. (2009) | 0 | 1 | 0 | 0 | 0 | 1 |
| Naçar et al. (2015) | 0 | 0 | 0 | 1 | 0 | 1 |
| Najafizadeh et al. (2009) | 0 | 1 | 0 | 0 | 0 | 1 |
| Ohwaki et al. (2006) | 0 | 1 | 0 | 1 | 0 | 2 |
| Ríos et al. (2019) | 1 | 1 | 1 | 1 | 1 | 5 |
| Rydzewska et al. (2018) | 0 | 0 | 0 | 0 | 0 | 0 |
| Sağiroğlu et al. (2015) | 0 | 1 | 0 | 1 | 0 | 2 |
| Sahin and Abbasoglu (2015) | 0 | 1 | 1 | 0 | 0 | 2 |
| Sampaio et al. (2020) | 0 | 0 | 0 | 1 | 0 | 1 |
| Sanavi et al. (2009) | 0 | 0 | 0 | 1 | 0 | 1 |
| Sayedalamin et al. (2017) | 1 | 1 | 0 | 0 | 0 | 2 |
| Sebastián-Ruiz et al. (2017) | 0 | 1 | 0 | 0 | 1 | 2 |
| Tagizadieh et al. (2018) | 0 | 1 | 1 | 0 | 0 | 2 |
| Tuesca et al. (2002) | 1 | 1 | 1 | 1 | 1 | 5 |
| Tumin et al. (2016) | 1 | 1 | 0 | 1 | 1 | 4 |
| Verma et al. (2020) | 1 | 0 | 1 | 1 | 0 | 3 |
| Wu et al. (2020) | 1 | 0 | 1 | 1 | 0 | 3 |
| Zahmatkeshan et al. (2014) | 0 | 1 | 1 | 0 | 1 | 3 |
| Zhang et al. (2014) | 0 | 0 | 1 | 0 | 0 | 1 |
